# Cotton Seed Germination Under the Main Abiotic Stress: Physiological Mechanisms, Molecular Responses, and Agronomic Strategies

**DOI:** 10.3390/plants15182881

**Published:** 2026-09-21

**Authors:** Huan Yu, Fenghao Zhang, Xiaomin Luo, Haoyang Geng, Junxian Chen, Shuxun Yu, Tian Pan, Zhen Feng, Junfeng Cao, Mengyuan Yan

**Affiliations:** 1 College of Advanced Agricultural Sciences, Zhejiang A&F University, Hangzhou 311300, China; yuhuann@stu.zafu.edu.cn (H.Y.);; 2 Centre for Cell & Developmental Biology, State Key Laboratory of Agrobiotechnology, School of Life Sciences, The Chinese University of Hong Kong, Shatin, Hong Kong SAR, China; 3 Key Laboratory of Wheat Biology and Genetic Improvement in South Yellow & Huai River Valley, Ministry of Agriculture and Rural Affairs, College of Agronomy, Anhui Agricultural University, Hefei 230036, China

**Keywords:** cotton, seed germination, salt stress, drought stress, heat stress, cold stress, stress response mechanisms, molecular breeding

## Abstract

Cotton is a globally vital economic crop and the primary source of natural fiber. Continued world population growth keeps driving demand for cotton lint, while the increasing frequency of extreme weather events exposes cotton production to a range of abiotic stresses, including salinity, drought, and temperature extremes. As the very first step in plant development, the germination phase is particularly vulnerable to these stresses, which severely impair seed germination and seedling establishment through multi-faceted physiological and metabolic disruptions, ultimately compromising crop uniformity and yield potential. A thorough understanding of the underlying tolerance mechanisms holds significant potential for improving cotton germination performance under adverse conditions. However, the precise functions of stress-responsive genes and their regulatory networks during cotton germination remain largely unresolved, which restricts the targeted genetic improvement of stress resilience in breeding programs. Here we systematically review the impacts of these stresses on cotton germination and the morphological, physiological, and molecular pathways underlying stress tolerance, together with current mitigation strategies. Future research should integrate multi-omics technologies, real-time phenotyping, and controlled-environment simulations to elucidate the signal–transcription–metabolism–phenotype regulatory pathways operative during germination, thereby providing a theoretical basis for breeding stress-resilient cotton cultivars and optimizing precision sowing technologies.

## 1. Introduction

As the most important fiber crop globally, cotton contributes an economic value of at least 600 billion dollars each year [1]. However, its sustainable production is increasingly constrained by abiotic stresses such as salinity, drought, and extreme temperatures, alongside growing land-use competition between grain and cotton cultivation [2]. These pressures have propelled the development of stress-resilient, high-yield cotton cultivars to the forefront of modern agricultural innovation. Within the entire crop production cycle, seed germination represents the foundational transition that determines when and under what environmental conditions a plant initiates its life cycle [3,4,5]. This process unfolds through three contiguous phases: an initial rapid imbibition that hydrates and softens the seed, a subsequent lag phase during which respiration surges and stored reserves are mobilized, and a final burst that ends when the radicle breaches the coat and the hypocotyl emerges through the micropyle [6]. As global climate change intensifies, marked by rising temperatures, altered precipitation patterns, and increased frequency of extreme weather events [7], germinating seeds must navigate increasingly hostile environments to successfully transition from heterotrophic dependence to photosynthetic autotrophy.

Successful germination emerges from the interplay between internal physiological constraints and external environmental cues. Internally, germination depends on the dynamic equilibrium between the growth potential of embryonic hypocotyls and the mechanical resistance exerted by the seed’s protective layers, including the endosperm, seed coat, and pericarp [8]. The seed coat primarily consists of cellulose, lignin, and various other compounds. Structural modifications such as elevated lignin or calcium deposits in the seed coat can induce hard-seededness, restricting water uptake and imposing physical dormancy [9,10,11]. Endogenous metabolites within seeds regulate the physiological processes of germination through intricate regulatory networks; for example, the accumulation of proanthocyanidins in the seed coat inhibits seed germination [12,13]. Plant hormones constitute the most pivotal internal regulators: abscisic acid (ABA) predominantly inhibits germination, whereas gibberellin (GA) promotes it, with their antagonistic interaction governing dormancy release [14]. Ethylene (ET) facilitates seed germination, particularly in seeds sensitive to ABA, by alleviating dormancy through the reduction in ABA levels or the enhancement of GA signaling pathways [15]; while auxin (IAA), jasmonic acid (JA), cytokinin (CTK), salicylic acid (SA), and brassinosteroids (BRs) exert indirect modulatory effects on seed dormancy and germination through crosstalk with ABA and GA pathways [16,17,18].

Externally, germination is shaped by both the parental environment during seed development and the immediate conditions during imbibition [19,20]. Abiotic stresses during germination prove particularly detrimental, as salinity and drought reduce water potential gradients, impeding the water uptake essential for metabolic activation [21]. Consequently, germination rates (refers to the speed or rapidity with which seeds germinate, often quantified as the time required for a certain proportion of seeds to germinate) decline, leaf development stalls, and final yield and quality deteriorate [22]. Upon radicle emergence, the seedling confronts direct environmental exposure, triggering rapid morphological and physiological adjustments [23]. Plant adaptation to abiotic stresses involves coordinated physiological, biochemical, and molecular mechanisms rather than isolated responses, making germination-stage stress tolerance a critical research priority for agricultural sustainability. For cotton specifically, these challenges are exacerbated in semi-arid and arid regions where soil salinization, spring drought, and extreme temperatures increasingly jeopardize seed germination and seedling establishment [24,25,26]. Impaired water imbibition and delayed developmental milestones lead to reduced, non-uniform emergence rates (hypocotyl with cotyledons breaking through the soil surface), creating a critical bottleneck that severely constrains overall yield potential [27].

Despite the agricultural importance of cotton seed germination, our mechanistic understanding of its stress responses remains surprisingly fragmented. The vast majority of studies have concentrated on post-emergence stages, generating detailed maps of the ABA pathway (such as *GhANN1*, *GhTOPP4aD*) [28,29], ion transporters (such as *GhNHX1*, *GhHKT1*) [30], antioxidant enzymes (such as *GhSOD*, *GhCAT*) [31] and heat shock proteins (such as *GhHSP70-26*) in leaves and roots [32], alongside multiple QTL hotspots for stress tolerance [33,34]. By contrast, the critical window from dry seed through imbibition to radicle protrusion is still described only in terms of cotton seed germination percentage (the proportion of seeds that successfully sprout under test conditions) and vigor indices; dynamic dissection of osmotic sensing, Ca^2+^ bursts, hormone rebalancing and the energy-metabolism switch from storage reserves to active growth is lacking, and an integrated signal–transcript–metabolism–phenotype roadmap is still missing. Neglecting stress tolerance mechanisms at the germination stage has become a bottleneck for achieving uniform, full and vigorous seedling establishment and, consequently, high and stable yield. Here, we systematically review cotton seed germination under three representative abiotic stresses, including salinity, drought, and extreme temperatures, with emphasis on their phenotypic effects, defense response patterns, key gene regulatory pathways, and management strategies for improving germination efficiency. This work aims to provide a theoretical foundation for stress-resilient molecular design and precision sowing practices.

## 2. Advances in Salt-Stress Resistance During Cotton Seed Germination

Soil salinization currently represents a critical ecological and environmental issue confronting the global community. The escalation of soil salinity has resulted in a substantial decline in the availability of arable land worldwide. According to data from the FAO, over 1381 million hectares of soil, equivalent to approximately 10.7% of the Earth’s terrestrial surface, are impacted by salinity (https://doi.org/10.4060/cd3044en). Compounded by freshwater scarcity, this trend intensifies the global tension between cash crop cultivation and food security priorities. Cotton presents a strategic solution to this dilemma. As a vital source of natural fibers for the textile industry, cotton demonstrates the capacity to grow in moderately saline regions where most food crops fail [35]. Through long-term management, cotton, as a pioneer crop for improving saline–alkali land, can reduce the salt content of saline–alkali soil by combining reasonable measures such as mulched drip irrigation and stubble management [36,37], thereby reducing root-zone salt stress and creating conditions for subsequently growing more salt-sensitive crops. Thus, breeding and deploying salt-tolerant cotton is not simply a strategy to expand fiber production; it is also a low-cost, biologically driven reclamation tool that converts saline wastelands into arable assets with long-term food security value.

### 2.1. Impacts of Salt Stress on Cotton Seed Germination

**Figure 1:**
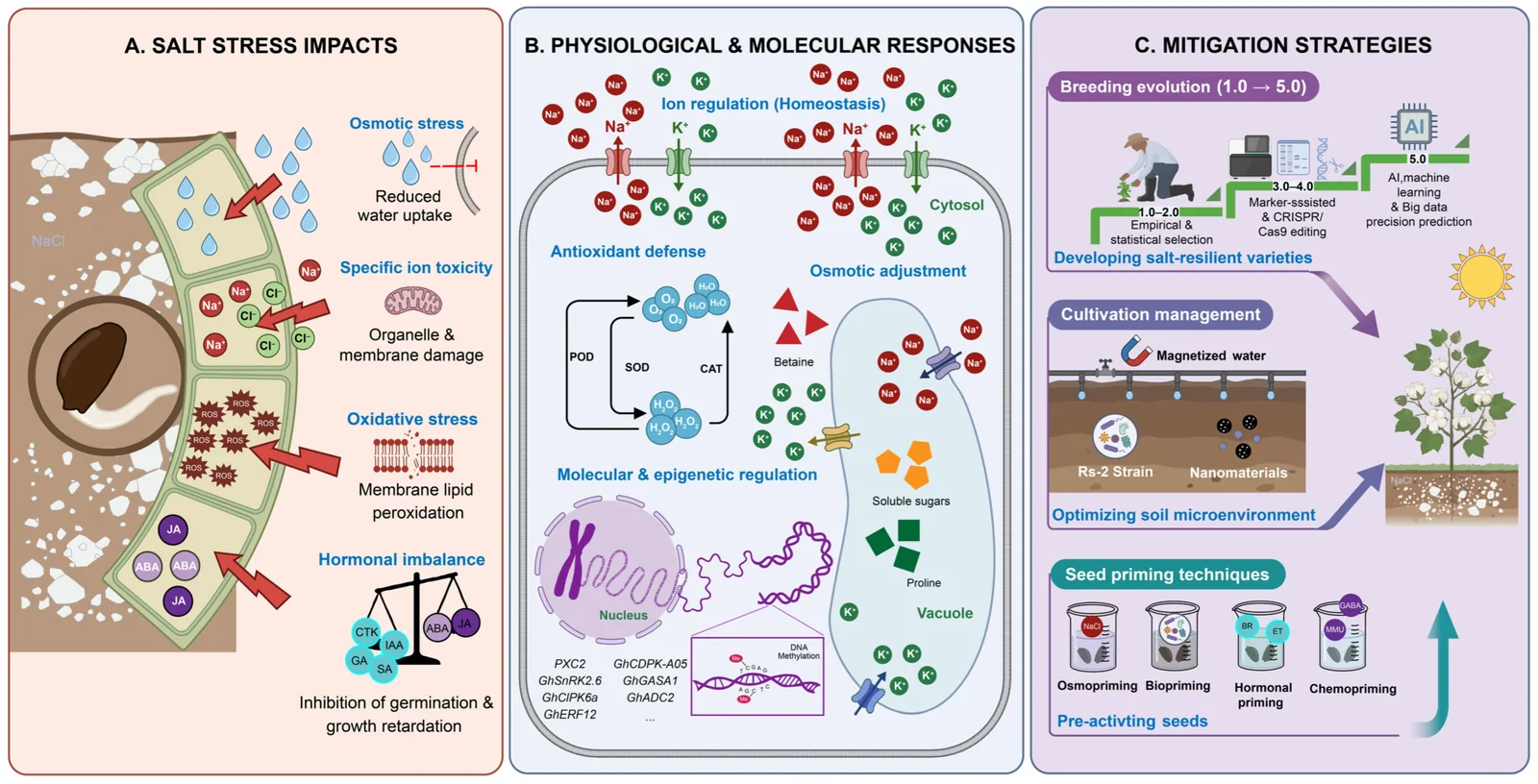
Mechanisms of salt stress inhibition and multi-faceted mitigation strategies in cotton seed germination. (**A**) Salt stress impedes cotton seed germination through osmotic stress, specific ion toxicity, oxidative stress, and hormonal imbalance. ROS, reactive oxygen species; JA, jasmonic acid; ABA, abscisic acid; CTK, cytokinin; IAA, indole-3-acetic acid; GA, gibberellin; SA, salicylic acid. (**B**) Physiological and molecular defense responses in cotton seeds under salt stress. POD, peroxidase; SOD, superoxide dismutase; CAT, catalase. (**C**) Advances in breeding technologies, cultivation management and seed priming strategies for enhancing salt tolerance. BRs, brassinosteroids; ET, ethylene; GABA, gamma-aminobutyric acid; MMU, monomethylolurea.

While cotton possesses moderate salinity tolerance during vegetative growth, it exhibits heightened sensitivity to salt stress during seed germination compared to later growth stages [38]. Recent studies have extended the evaluation window to cover the entire process from radicle protrusion through the seed coat to plumule elongation under abiotic stress treatment [39,40,41]. Salt stress markedly reduces germination success and frequently prevents seedling establishment, rendering this stage a critical bottleneck in salt-tolerance breeding [42,43]. The detrimental effects of salinity operate through three pathways (Figure 1A), namely osmotic stress, specific ion toxicity, and oxidative stress [25,44]. These pathways do not act in isolation but constitute an interconnected system of damage that collectively disrupt the germination program. Osmotic stress reduces water availability during the absorption process as external osmotic potential rises, directly constraining embryo expansion and radicle emergence. Moreover, this water deficit triggers secondary stresses, including reduced metabolic activity and impaired mobilization of stored reserves. Concurrently, the accumulation of specific ions such as sodium and chloride disrupts enzyme structure and function, damages organelles and plasma membranes, and interferes with protein synthesis, energy production, and respiration [45]. Critically, ion toxicity and osmotic stress synergize to reduce water uptake, which limits ion dilution, while ion accumulation further inhibits water absorption, creating a self-amplifying cycle. Furthermore, salt-induced metabolic disruption elevates reactive oxygen species (ROS) production, overwhelming the antioxidant capacity that normally maintains redox homeostasis [45,46]. Excessive ROS cause cross-linking of biomolecules and membrane damage, leading to increased lipid peroxidation, malondialdehyde (MDA) content, and electrolyte leakage [46]. The loss of membrane integrity due to oxidative damage likely serves as the terminal event that renders cells nonviable. Hormonal reprogramming integrates these physical and chemical stresses into a coherent physiological response. Upon perceiving salt stress signals, the hormonal balance in seeds shifts toward stress adaptation, with growth-promoting hormones (such as GA) declining while stress-related hormones (such as ABA) accumulating [47,48,49]. Thus, this transition prioritizes stress survival over growth promotion, arresting germination under severe conditions. In summary, the convergence of osmotic, ionic, and oxidative damage on cellular homeostasis, manifested as reduced water availability, blocked mobilization of stored reserves, and disruption of biological macromolecule organization and content, rather than any single mechanism, determines germination failure; therefore, identifying which node offers the greatest leverage for genetic improvement is essential for targeted breeding.

### 2.2. Response of Cotton Seeds to Salt Stress During Germination

The response of cotton seeds to salt stress during the germination represents a hierarchically organized adaptive system that operates across molecular, metabolic, and morphological levels (Figure 1B). Rather than isolated protective reactions, these responses constitute an integrated network centered on energy homeostasis and osmotic balance. At the physiological level, salt-tolerant varieties (e.g., Xinluzhong 82, Xinluzhong 68) employ a conservative strategy during early imbibition [50], actively reducing water uptake rates to minimize passive salt influx while maintaining cellular osmotic potential against external low water potential. This initial restraint is complemented by metabolic reprogramming, specifically decelerating total sugar and protein catabolism to prolong energy release duration, coupled with early accumulation of crude fat as concentrated energy reserves for germination. Morphologically, the trade-off between root longitudinal growth inhibition and radial thickening preserves both absorptive surface area and mechanical anchorage under stress. These physiological and metabolic adjustments are reinforced by three coordinated defense mechanisms. First, compatible substances such as soluble sugars, proline, and betaine rapidly accumulate, accompanied by an increase in K^+^ content, which decreases seed osmotic potential and maintains the imbibition driving force [38]. Second, ionic homeostasis operates through Na^+^ efflux, sequestration, and restricted transport, ensuring that core germination tissues maintain high K^+^/Na^+^ ratios and normal metabolic functions [38]. Third, antioxidant defense responds to salt-induced ROS bursts with synchronous up-regulation of superoxide (SOD), peroxidase (POD), and catalase (CAT), effectively scavenging ROS and reducing MDA content to preserve cell membrane integrity [51]. Hormonal crosstalk, particularly the ABA-GA antagonism modulated by salt-activated catabolic enzymes [52], likely serves as the systemic coordinator of these responses. The efficiency of coordination among energy management, osmotic maintenance, and oxidative protection, rather than the magnitude of any single response, appears to distinguish salt-tolerant from sensitive genotypes.

Translating these physiological strategies into molecular terms, current studies have begun to map the regulatory architecture underlying salt tolerance during cotton seed germination. However, progress remains fragmented: while numerous genes have been identified through gene family analysis, homologous cloning, and omics approaches, functional validation in cotton itself remains scarce, with most studies relying on heterologous expression systems to demonstrate effects on germination rate or vigor (Table S1), leaving the native regulatory pathways and mechanisms within cotton largely unresolved. This paucity of in planta evidence, including knockout, overexpression, or complementation assays in the cotton native genetic background, precludes the construction of reliable regulatory networks on seed germination under salt stress, as protein–protein interactions, spatiotemporal expression patterns, and pathway hierarchies cannot be confidently inferred from heterologous data alone. These limitations underscore the urgent need for genetic engineering approaches to validate candidate genes and establish their breeding utility.

To make sense of these disparate findings, we synthesize evidence from the limited set of functionally characterized cotton genes into a perception–transduction–execution hierarchical model. At the perception layer, membrane-localized receptor-like kinases serve as upstream sensors of salt stress. *PXC2*, a leucine-rich repeat receptor-like kinase (LRR-RLK), has been identified as a key candidate gene through transcriptomic profiling of contrasting cotton varieties under salt stress [53], though its specific molecular mechanisms within cotton germination remain to be elucidated. The transduction layer integrates multiple signaling streams. The SnRK2 family, particularly *GhSnRK2.6*, positively regulates salt tolerance during cotton seed germination [54], and likely functions within ABA signaling pathways to integrate and relay environmental stress cues [55]. *GhCIPK6a*, identified via homology-based cloning, coordinates multiple downstream pathways including ion homeostasis, antioxidant defense, and MAPK cascades [56]. Moreover, both the *GhSnRK2.6* and *GhCIPK6a* genes have been evaluated for salt tolerance during the germination stage in cotton plants (Table S2). The ethylene response factor *GhERF12*, uncovered through transcriptome sequencing of primary roots from salt-tolerant LMY37 and salt-sensitive ZM12 after 24 h salt stress, modulates ROS levels and antioxidant enzyme activities [57]. Intriguingly, the calcium-dependent protein kinase *GhCDPK-A5*, identified via combined recombinant inbred line (RIL) population analysis and transcriptome sequencing, shows significant down-regulation in tolerant lines [58]. This suggests potential negative regulatory nodes that fine-tune response intensity, reflecting a recurring theme in stress signaling where suppression of inhibitory pathways may be as critical as activation of protective ones. The execution layer encompasses functional effectors such as ion transporters, antioxidant enzymes, and osmoprotectant biosynthetic enzymes that directly implement cellular adaptation [59]. Beyond the core signaling hierarchy, epigenetic mechanisms provide an additional regulatory dimension that may confer stress memory [60]. Differential DNA methylation in genes related to hormone pathways (e.g., IAA, GA, BR) and cell elongation has been detected in salt-stressed hypocotyls [39], suggesting that chromatin-level modifications contribute to transcriptional reprogramming during germination. Such epigenetic marks potentially serve as somatic or transgenerational stress memories [61], though their stability and heritability in cotton require experimental validation.

From a genetic architecture perspective, salt tolerance during germination behaves as a complex quantitative trait controlled by multiple minor-effect loci rather than major genes. QTL mapping and genome-wide association study (GWAS) approaches have identified several genomic regions associated with germination vigor under salt stress, including the *qRGR-A04-1* locus harboring *GhGASA1* and *GhADC2*, which negatively regulate tolerance via GA and phosphatidic acid signaling [62], as well as additional loci reported on chromosomes A04, A05, and others [58,62,63,64,65,66] (Table 1). However, a critical gap remains in that most candidate genes await functional validation, and the allelic diversity underlying natural variation in salt tolerance has not been systematically characterized. Moreover, the relationship between these mapped loci and the signaling modules described above is largely unknown, precluding the construction of genotype-to-phenotype predictive models. To bridge this gap, future efforts should prioritize large-scale screening and functional validation of germination-specific salt tolerance genes, clarifying their roles within the salt tolerance regulatory network and their allelic contributions to natural variation. Such advances will translate genetic data into actionable breeding resources, enabling pyramiding of favorable alleles and development of salt-resilient cotton cultivars.

### 2.3. Mitigation Strategies During Seed Germination Under Salt Stress

To significantly enhance salt tolerance of cotton seeds during germination, researchers have developed a multi-pronged strategy portfolio encompassing genetic improvement, cultivation environment modification, and physiological preconditioning (Figure 1C). Genetic improvement has undergone iterative technological revolutions. The initial phase of crop breeding, known as Breeding 1.0, was characterized by its dependence on practical experience and the accumulated wisdom passed down by generations of farmers and pioneering breeders. Despite its slower pace, this early phase established the fundamental principles that all subsequent technologies would refine and extend. Subsequently, Breeding 2.0 was pioneered following the identification of Mendelian inheritance and the emergence of quantitative genetic theory. Representing a shift toward scientific rigor, this phase harnessed genetic and statistical principles to methodically enhance crossbreeding, experimental field tests, and the selection of desirable characteristics [67]. The advent of Breeding 3.0 was propelled by transformative advances in molecular biology, particularly DNA marker-assisted selection and genome sequencing. This shift enabled agriculture to enter a new age defined by precision at the molecular scale [68]. In the Breeding 4.0 phase, the deep integration of genetic engineering and big data emerged as a new norm in the breeding field. With genome editing technologies, such as CRISPR/Cas9, breeders can perform precise manipulations and regulation on plant genomes, thereby dramatically accelerating the overall efficiency of the breeding process [69]. The emerging Breeding 5.0 phase harnesses artificial intelligence, including machine learning and deep learning, to effectively extract patterns from massive genomic and phenotypic datasets, enabling more precise selection decisions and accelerated varietal development [70]. The transformative journey from Breeding 1.0 through 5.0 embodies the ongoing technological progression in crop enhancement [68], with each phase of innovation providing robust support for the breeding of salt-tolerant cotton varieties. Looking ahead, as current functional validation in cotton remains limited, leaving most candidate genes from mapping and omics studies unconfirmed, realizing the full potential of Breeding 4.0 and 5.0 requires prioritized efforts to identify and characterize more regulatory genes specifically governing salt stress responses during germination. Gene pyramiding has emerged as a key strategy to integrate multiple beneficial traits into elite varieties [71]. Through advanced genetic engineering, validated germination-specific salt tolerance genes can be pyramided with yield and fiber quality loci, enabling the breeding of high-yielding, premium fiber varieties with multi-stress resilience.

Besides genetic improvement, cultivation management methods provide immediate, practical solutions by directly optimizing the soil microenvironment and field conditions for seed germination under salt stress (Figure 1C). A single experiment conducted in Xinjiang found that cotton seeds treated with 3000 GS magnetized brackish water showed significantly higher final emergence rate, emergence index, and vigor index than those treated with brackish water [72]. Similarly, microbial embedding offers biological solutions, such as the Rs-2 strain, which improves germination under salt stress by maintaining ROS homeostasis and reducing membrane lipid peroxidation damage [73]. In recent years, nanopriming with nanomaterials (e.g., ZnO, Ag, Fe, or Si nanoparticles) offers a safer and eco-friendly approach to enhance crop stress tolerance by activating germination-related metabolic processes, including enhanced aquaporin expression, amylase activity, and antioxidant capacity [74]. Nanopriming with carbon dots significantly increased cotton seed germination percentage, seed vigor index, and seedling root length under salt stress compared with water-primed controls [75].

In addition to the genetic improvement and cultivation approaches, seed priming offers a third, immediately deployable tier of intervention (Figure 1C). This economical hydration technique promotes rapid germination and uniform seedling emergence by physiologically pre-activating seeds before they encounter saline conditions [76]. Depending on the priming agent used, common priming methods include hydropriming, osmotic priming, hormonal priming, and biopriming with microorganisms (Table S3). These priming techniques enhance cotton seed germination under salt stress by shortening the imbibition period, activating earlier germination-related enzymes, generating metabolites, and improving osmotic regulation. Priming also induces a range of molecular changes, such as de novo synthesis of DNA and proteins, DNA repair, increased ATP production, or accumulation of osmolytes and antioxidant metabolites [77]. Distinct from breeding and cultivation, priming requires no genetic alteration or extensive field inputs, making it particularly valuable for resource-limited farmers facing immediate salinity challenges. However, this convenience comes with trade-offs. The optimal priming protocol varies by cultivar, salinity level, and environmental conditions [78], complicating standardized recommendations. Consequently, priming functions best as a complementary tool bridging genetic potential and environmental reality, rather than a standalone solution.

These three strategy tiers operate at different time scales and resource intensities yet share a common mechanistic target, namely maintaining cellular energy availability, ionic homeostasis, and membrane integrity during the critical germination window. Future integration of these approaches, such as priming genetically superior seeds under optimized soil conditions, may achieve synergistic outcomes unattainable by any single strategy. The overarching goal is to shift from reactive damage mitigation to proactive resilience engineering, ensuring cotton establishment in increasingly saline production environments.

## 3. Advances in Drought-Stress Resistance During Cotton Seed Germination

The degradation of the global environment has led to drought stress emerging as a predominant abiotic factor constraining crop productivity. The United Nations Convention to Combat Desertification (UNCCD) reports global drylands expanded by about 4.3 million km^2^, an area equal to half the size of the continent of Australia/Oceania to cover more than 40 percent of global land (excluding Antarctica) (https://doi.org/10.18356/9789295128163). With the expansion of semi-arid conditions, important crop growing areas are facing a grim future, becoming the largest single driver of global agricultural system degradation [79]. Water is an important component of plants and is indispensable for cell growth and development [80]. It maintains the cell structure and serves as an important medium for various growth processes. Drought is characterized by the depletion of soil moisture due to insufficient rainfall or water retention capacity, which ultimately affects crop yields [81]. Drought stress arises when water loss from plants exceeds the absorption capacity of their roots, resulting in a reduction in water content to levels that impair normal physiological functions. Even after the cessation of drought conditions, plants may require extended periods, ranging from months to years, to repair root damage and regain their growth potential [82]. Global water shortage has become a major and urgent issue in agricultural development, seriously threatening the growth and development of crops. Rational utilization of the drought resistance ability of crops is an important, effective, and economical measure to alleviate water shortage and ensure the sustainable development of agriculture.

### 3.1. Impacts of Drought Stress on Cotton Seed Germination

Drought stress profoundly impacts plants throughout the entire process of seed germination and early growth, wherein water plays an indispensable role in seed activation and root system establishment. Numerous studies have demonstrated that drought stress significantly suppresses key indices such as germination potential (the percentage of normally germinated seeds at the germination peak, typically assessed over the first one-third of the test period), germination rate, and radicle elongation [83,84]. However, previous research has predominantly concentrated on the critical yield-forming stages of cotton, namely the seedling and boll development phases [85], while comparatively neglecting the germination stage. This gap likely originates from the relatively short duration of the germination period, which constrains the detection window, and from researchers’ greater emphasis on growth stages that directly determine final yield. Nevertheless, emerging evidence suggests that the germination stage plays an important role in establishing drought resistance capacity throughout the entire growth cycle [76]. Since the germination stage and later-stage drought resistance involve distinct genetic regulatory mechanisms, and germination vigor may exert significant effects on subsequent growth performance [84], investigating the drought resistance of cotton germplasm during the germination stage is equally essential.

**Figure 2:**
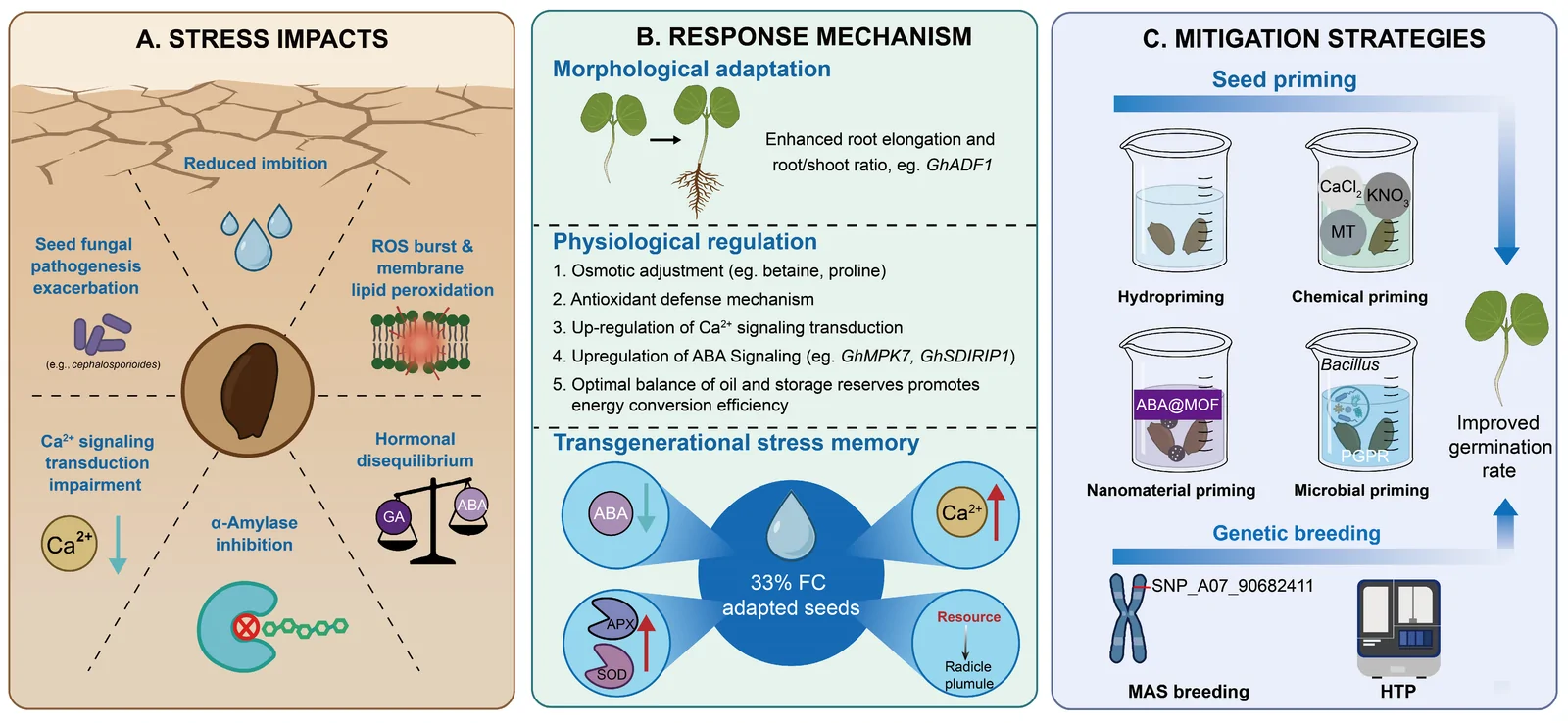
A comprehensive framework illustrating the responses of cotton seeds to drought from physiological impacts and molecular mechanisms to strategic interventions. (**A**) Drought stress impedes cotton seed germination through water deficit, oxidative damage, hormonal imbalance, calcium signaling disruption, nutrient mobilization blockage, and pathogen susceptibility. (**B**) Within-generation reactive and transgenerational predictive defense responses in cotton seeds under drought stress. FC, field capacity; APX, ascorbate peroxidase. (**C**) Advances in seed priming and genetic strategies for enhancing drought tolerance. MT, melatonin; PGPR, plant growth-promoting rhizobacteria; MAS, marker-assisted selection; HTP, high-throughput phenotyping.

Cotton seeds exhibit high water demand during germination, rendering them particularly vulnerable to soil moisture deficits [84]. Extreme drought stress inhibits cotton seed germination through interconnected mechanisms spanning physical barriers, hormonal reprogramming, metabolic collapse, and compromised pathogen defense (Figure 2A). At the physical perception level, impaired imbibition disrupts osmotic balance and cellular turgor, directly blocking cell expansion required for radicle protrusion. Simultaneously, drought-induced tightening of the seed coat increases mechanical resistance, creating a dual physical barrier that arrests embryo growth even before metabolic inhibition manifests [84]. At the signaling integration level, drought stress triggers a hormonal switch that reconfigures germination programming. The dynamic equilibrium between GA and ABA, the core hormonal axis regulating seed germination and seedling establishment [85], is systematically disrupted, with ABA accumulation reinforcing seed coat impermeability and inducing secondary dormancy, while impaired GA biosynthesis inhibits radicle elongation [85]. The hormonal reprogramming is compounded by impaired calcium-mediated signal transduction, evidenced by reduced seed calcium reserves in drought-sensitive cotton seeds under drought stress [86]. The signaling impairment compromises the seed’s ability to coordinate adaptive responses, thereby amplifying the decline in seedling vigor beyond what hormonal arrest alone would cause. At the metabolic level, drought stress precipitates dual collapse in which ROS burst overwhelms antioxidant defenses, evidenced by suppressed SOD and ascorbate peroxidase (APX) activities and escalated MDA accumulation, leading to membrane lipid peroxidation and cellular integrity loss [85,86]; concurrently, α-amylase activity suppression decelerates starch mobilization, creating an energy bottleneck that restricts both germination completion and early seedling growth [85]. Beyond these direct physiological disruptions, drought stress compromises the seed’s innate defense capacity, creating a secondary vulnerability cascade characterized by significantly elevated infection rates by *Colletotrichum gossypii* var. *cephalosporioides*, with the degree of infection intensifying as stress duration prolongs, potentially due to increased membrane permeability or reduced defense enzyme activity (e.g., chitinase) [87]. The pathogen–drought interaction introduces a biological contingency into germination outcomes, whereby even seeds with robust physiological drought tolerance may fail to establish if their defense systems are compromised by drought precisely when pathogen infection occurs. But this contingency is rarely integrated into laboratory-based drought tolerance assessments.

Collectively, drought stress delays germination kinetics, reduces emergence uniformity, weakens seedling vigor, and restricts biomass accumulation through interconnected physical, hormonal, metabolic, and defensive mechanisms. A critical insight that emerges from this hierarchical framework is that single-target interventions, such as antioxidant supplementation alone, are likely insufficient, as upstream signaling blocks (hormonal arrest) and physical barriers (impaired imbibition) remain intact. Moreover, the seed coat’s dual role as both physical barrier and pathogen entry point suggests that breeding for optimized permeability, rather than simply reduced thickness, could reconcile the trade-off between germination speed and disease resistance, a hypothesis that warrants direct empirical testing.

### 3.2. Drought Stress Response in Cotton Seed During Germination

The response of cotton seeds to drought stress during germination has been characterized by two seemingly distinct phenomena: the threshold effect describing dose-dependent physiological plasticity within a generation, and the memory effect capturing transgenerational epigenetic adaptation. Rather than treating these as separate phenomena, we propose an integrated biphasic adaptation strategy framework that positions within-generation plasticity and transgenerational memory as complementary evolutionary solutions to temporal uncertainty in water availability. This reconceptualization reveals that cotton seeds deploy reactive adaptation when stress occurs during their own germination (costly but necessary for immediate survival) and predictive adaptation when maternal plants pre-condition progeny for anticipated stress (metabolically efficient but requiring multi-generation selection). Understanding the mechanistic basis and ecological trade-offs of these distinct phases is essential for designing breeding and agronomic interventions that match the appropriate adaptive strategy to target environments.

Phase I: Within-Generation Reactive Adaptation

When cotton seeds encounter drought during germination, they construct a morphological–physiological–molecular response network that operates in a dose-dependent manner until a critical physiological threshold is exceeded. At the level of morphological adaptation (Figure 2B), the plastic adjustment of root architecture is the core strategy. Under mild to moderate stress, cotton promotes the elongation of the main root and significantly increases the root/shoot ratio. This expands the water absorption area while reducing transpiration water consumption in the aerial part, thus optimizing water use efficiency [83]. This plasticity is genetically regulated: silencing of the actin depolymerizing factor *GhADF1* may enhance radicle breakthrough capacity by modulating cytoskeletal dynamics, thereby accelerating germination completion and seedling establishment under water deficit [88]. However, this strategy exhibits clear limitations, as once stress exceeds the critical threshold, the loss of cell turgor will impede root elongation [89]. This threshold constraint implies that breeding for extreme drought environments may require shifting selection targets from plasticity per se to mechanisms that extend the functional range of plastic responses.

Complementing these morphological adaptations, physiological mechanisms provide essential protection at the cellular level. The active accumulation of osmotic adjustment substances constitutes the core adaptation strategy. Drought stress induces cotton seeds to synthesize large amounts of osmotic adjustment substances such as proline, soluble sugars, and soluble proteins, which reduce cellular osmotic potential to enhance the water absorption ability, stabilize protein structures and maintain metabolic activity, ultimately improving radicle cells survival under water shortage [90]. Meanwhile, seeds deploy antioxidant defense mechanisms, including the regulation of antioxidant enzyme activities and non-enzymatic antioxidants, to mitigate ROS-induced oxidative damage [90]. Importantly, numerous genes validated in heterologous systems (Table S1) appear to function through coordinated osmotic and antioxidant pathways, though their direct roles in cotton remain largely unconfirmed. In addition, calcium signaling integrates these responses by functioning as a second messenger that acutely senses water deficit. Under drought stress, intracellular Ca^2+^ surges activate kinase pathways such as CDPK and SnRK2, thereby strengthening stress signal transduction and enabling seeds to mount rapid adaptive responses [91]. In addition, drought stress activates ABA signaling during germination, thereby enhancing stress tolerance. Gao et al. (2025) [92] showed that *GhMPK7* enhances ABA signaling by phosphorylating and promoting the degradation of GhSDIRIP1, a negative regulator of the ABA pathway. Their study further revealed that *GhMPK7*-silenced seeds displayed reduced ABA sensitivity, exhibiting accelerated germination and increased root length under ABA treatment, yet showed compromised germination performance and reduced root elongation under mannitol-simulated drought stress [92] (Table S2). The pivotal role of ABA extends beyond drought, as this hormone serves as a master regulator of abiotic stress responses by orchestrating stomatal closure, osmotic adjustment, and gene expression reprogramming across multiple stress scenarios including salinity, cold, and heat. Adding complexity to these adaptive strategies, an intriguing metabolic trade-off emerges in which high oil content accelerates cotton seeds germination under optimal conditions but suppresses performance under drought and salt stress owing to impaired fatty acid enzyme activity and energy supply [93]. This metabolic vulnerability suggests that germination speed and stress resilience represent competing selection objectives in cotton breeding programs, and that optimizing the efficiency of energy conversion from storage reserves, rather than merely reducing oil content, may offer a pathway to reconcile these competing demands.

Phase II: Transgenerational Predictive Adaptation

Significantly, transgenerational stress memory can systematically reshape the physiological state of progeny seeds [94], enabling proactive preparation rather than reactive response. Cotton seeds from maternal plants experiencing 33% field water holding capacity stress for three consecutive years exhibit enhanced adaptive effects, with significantly improved germination ability [86] (Figure 2B). This adaptive reconstruction operates through multilevel collaboration of optimized endogenous hormones, enhanced signal transduction, and pre-activation of the defense system. Specifically, stress memory seeds maintain a low ABA content while promoting the large-scale accumulation of calcium ions to strengthen stress signal transduction systems for rapid activation. Concurrently, the antioxidant system shows a pattern of leaves protecting seeds, that is, the activities of SOD and APX in maternal leaves are significantly upregulated, effectively reducing the damage of ROS to the embryo and pre-activating the seed antioxidant system. Furthermore, the resource allocation strategy is optimized, with more assimilates being preferentially supplied for the elongation of the radicle and plumule rather than for stress defense, and this advantage during the germination period can be extended to the yield formation stage. The transgenerational transmission of various abiotic stress memory in plants has been reported to be mediated by epigenetic and/or non-epigenetic mechanisms [95]. At the epigenetic level, DNA methylation, histone modifications, and small RNA-directed pathways constitute the primary molecular basis for meiotically heritable memory across multiple generations [96,97,98]. At the non-epigenetic level, seed provisioning also contributes to intergenerational plasticity; environmentally stressed maternal plants are able to maintain or even increase the allocation of mRNAs, proteins, hormones, and nutrient reserves to developing seeds to maximize seedling survival [95].

These findings reveal a fundamental distinction: Phase I reactive adaptation incurs immediate metabolic costs and growth penalties, whereas Phase II predictive adaptation reduces these costs through anticipatory preparation but requires multi-generation stress exposure for full manifestation. Current breeding programs predominantly select Phase I plasticity in single-generation trials, potentially overlooking valuable Phase II predictive capacity. Moreover, the observation that stress memory stabilizes germination percentage at 100% with a super-strong vigor index [86] suggests that epigenetic programming may achieve phenotypic outcomes unattainable through genetic selection alone; such a prospect has profound implications for breeding design.

Synthesizing these insights, drought tolerance of cotton seeds during germination is governed by a series of mechanisms including root morphological plasticity, osmotic adjustment, antioxidant defense or hormone signal reprogramming, and modulated by transgenerational epigenetic memory that couples immediate inhibition with a memorized pre-adaptation; yet current studies remain largely descriptive. Although GWAS has identified four candidate genes (*RD2*, *PIP2*, *HAT22* and *PP2C*) through phenotyping hypocotyl length and germination percentage under drought stress, their regulatory roles and molecular mechanisms remain uncharacterized [99]. The predominant reliance on heterologous validation (Table S1) creates uncertainty about cotton-specific gene function and regulatory divergence. Priority research directions may therefore include: (i) large-scale screening of germination-stage drought-tolerant germplasm and identification of key genetic loci, coupled with multi-omics integration to elucidate molecular regulatory networks in cotton underlying drought resistance; (ii) combining high-resolution phenomics and genomics to quantify how drought intensity and duration reshape energy metabolism during radicle protrusion; and (iii) systematic characterization of drought memory effects across genotypes and establishment of seed pre-adaptive protocols to enhance germination performance under water deficit, thereby providing a design-oriented theoretical framework for breeding drought-resilient cotton.

### 3.3. Strategies for Mitigating Drought Stress During Seed Germination

Various seed priming techniques have proven effective in mitigating drought stress during cotton germination (Figure 2C), yet their underlying mechanisms differ fundamentally from genetic improvement strategies. Conventional hydropriming can directly alleviate osmotic stress by optimizing water imbibition [100], while chemical priming agents (such as CaCl_2_, KNO_3_, and glycine betaine) enhance membrane stability, cellular turgor and energy supply through the elevated accumulation of osmoregulatory substances (e.g., proline and soluble sugars) [101]. These treatments essentially mimic or pre-activate the seed’s intrinsic stress response pathways, for instance, melatonin priming activates antioxidant systems and hormonal balance regulation [102,103] that closely mirror the within-generation reactive adaptation mechanisms described earlier. This suggests priming may serve as a ‘physiological mimic’ of transgenerational memory, activating similar protective pathways within a single generation without requiring multi-generation stress exposure. More forward-looking are nanomaterial-based priming and microbial priming strategies. Specifically, carbon-based nanomaterials applied at 50 and 100 µg mL^−1^ increased cotton seed germination by 20% relative to the controls, whereas ABA@MOF at 150 mg L^−1^ significantly enhanced germination potential, reaching a maximum germination rate of 70.7%. These improvements may be attributed to enhanced aquaporin activity, improved nutrient-use efficiency, and modulated endogenous hormone balance in cotton seeds [104,105]. Similarly, inoculation with endophytes or phyllosphere bacteria enhances seed vigor and drought tolerance through multiple growth-promoting mechanisms, including phytohormone secretion (e.g., IAA), synthesis of osmotic substances (e.g., exopolysaccharides), and ACC deaminase expression [106,107]. However, priming effects are context-dependent, with optimal protocols varying across germination environments and seed lots. Such variability underscores why priming should complement, not substitute for, systematic breeding for drought tolerance.

Turning to genetic breeding, a structural paradox characterizes current research on cotton germination-stage drought tolerance. While gene function studies in heterologous systems (e.g., *Arabidopsis*, Table S1) offer convenient entry points for understanding conserved mechanisms, their direct applicability to cotton genetic improvement remains limited. The molecular marker SNP_A07_90682411 developed by Guo et al. (2025) provides a useful auxiliary selection tool, yet single markers inadequately capture the polygenic nature of drought tolerance [93]. The deeper issue lies in our systematic ignorance of cotton-specific drought tolerance pathways, namely genetic modules not conserved in model plants but evolved during cotton’s long-term adaptation to arid environments, which severely constrains modern biotechnology applications. Resolving this impasse requires leveraging high-throughput phenomics and artificial intelligence algorithms for large-scale rapid screening of drought-tolerant germplasm to accelerate elite allele mining, while simultaneously establishing cotton-native functional validation platforms to systematically characterize genes that remain silent or functionally divergent in heterologous systems. Finally, the synergy between agronomic practices and genetic improvement warrants careful consideration. Although plastic film mulching is widely adopted in Xinjiang’s cotton belt to alleviate immediate water stress, maintain soil temperature, and reduce the incidence of cotton diseases, it introduces significant environmental trade-offs. In particular, residual plastic accumulation (white pollution) degrades soil structure and microbial community composition, posing long-term sustainability challenges [107,108]. Thus, an ideal technology integration strategy would employ targeted seed priming and precision irrigation as transitional resilience measures, while prioritizing genetic improvement for intrinsic drought tolerance to ultimately reduce dependency on plastic mulch, thereby promoting the development of plastic-mulch-free cotton production systems. This three-dimensional genetics–environment–management framework may represent the essential pathway toward sustainable enhancement of cotton germination-stage drought tolerance.

## 4. Advances in High-Temperature Stress Resistance During Cotton Seed Germination

For over a century, human activities such as fossil fuel combustion, deforestation, and industrial production have continuously driven global warming. The Intergovernmental Panel on Climate Change (IPCC) noted in its Sixth Assessment Report that the global temperature rise is projected to reach 1.5 °C between 2021 and 2040 (https://www.ipcc.ch/report/sixth-assessment-report-cycle/, accessed on 22 April 2026). Against this backdrop, the frequency and intensity of extreme weather events like high temperatures and droughts have significantly increased. Heat stress refers to an abiotic stress condition in which environmental temperatures persistently or temporarily exceed the optimal range for plant growth, thereby adversely affecting plant development [109]. Most plants have an upper temperature tolerance limit of about 35 °C; exceeding this threshold leads to heat stress [110]. Based on duration, heat stress can be categorized into long-term (days to weeks) and short-term (minutes to hours). Regardless of duration, heat stress disrupts key physiological processes such as morphogenesis, photosynthesis, and respiration, ultimately resulting in reduced crop yield and quality degradation [111,112,113]. Therefore, enhancing plant tolerance to heat stress has become a critical and long-term scientific challenge requiring urgent attention.

### 4.1. Impacts of High-Temperature Stress on Cotton Seed Germination

**Figure 3:**
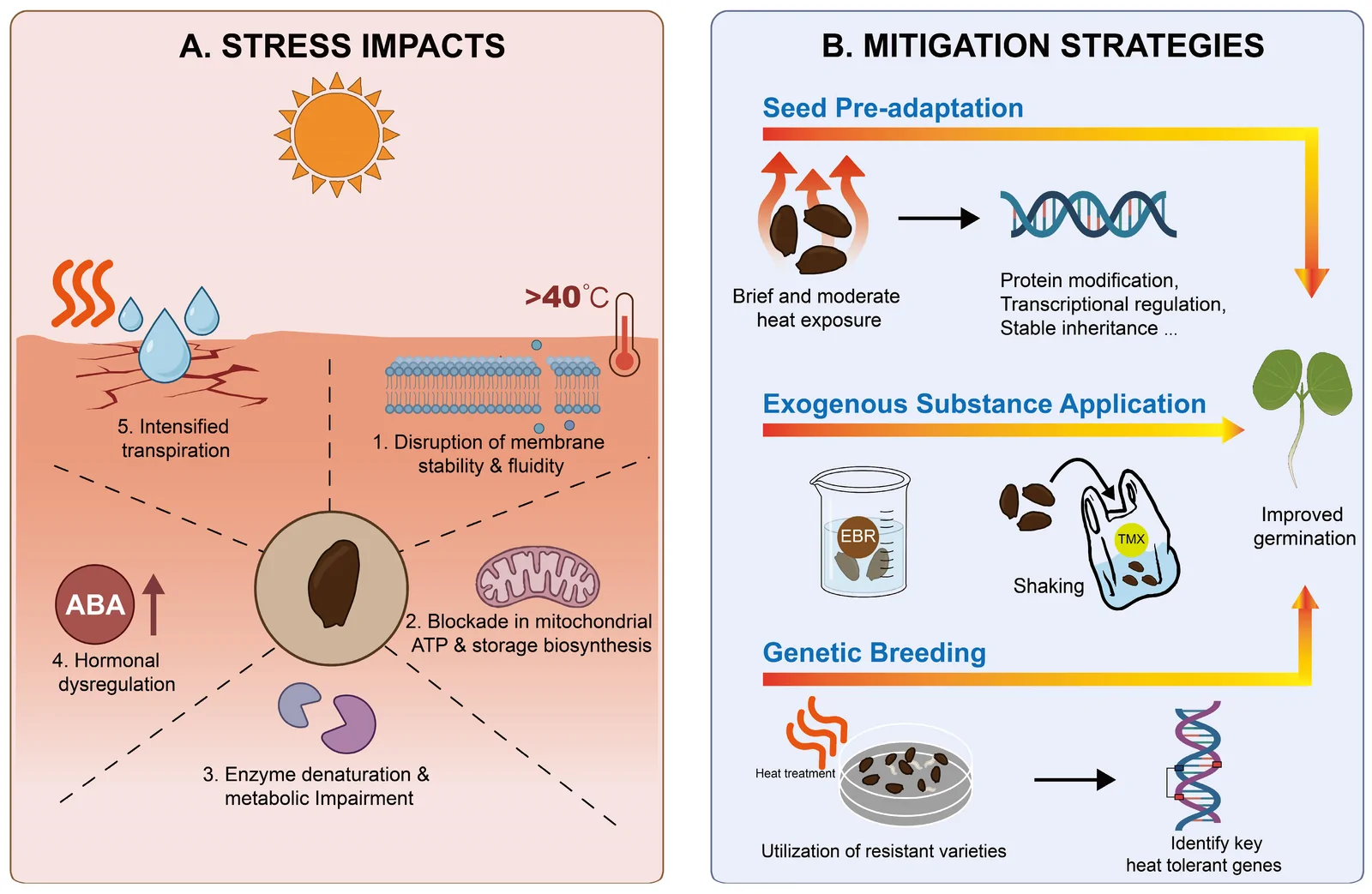
Heat stress impacts on cotton seed germination and multilevel mitigation strategies. (**A**) Mechanisms of heat damage. Temperatures exceeding 40 °C impair germination through five interconnected pathways, including membrane destabilization, mitochondrial ATP synthesis inhibition, enzyme denaturation and metabolic decline, ABA-driven hormonal imbalance, and excessive transpiration leading to water deficit. The arrow indicates an increase in ABA content. (**B**) Adaptive and intervention strategies. Three complementary approaches enhance thermotolerance, namely stress priming, chemical intervention, and genetic improvement. EBR, 24-epibrassinolide; TMX, thiamethoxam.

Major cotton-growing regions worldwide are experiencing varying degrees of sustained high temperatures. It has been reported that for every 1 °C increase in average temperature, cotton yield decreases by 1.64% [114]. Previous studies have found that high temperatures during flowering and budding stages reduce pollen viability [115], inhibit boll development, increase bud and boll shedding [116], and reduce fiber uniformity [117], ultimately compromising cotton yield and quality [118]. However, germination, a critical developmental stage highly sensitive to temperature, remains underexplored under heat stress. While optimal germination temperatures vary significantly among cotton varieties, most cotton seeds exhibit the best germination and seedling growth performance between 24 °C and 30 °C [119,120]. Research indicates that soil surface temperatures consistently exceeding 40 °C often result in germination failure or seedling death, a problem particularly prevalent in tropical production regions such as India and Pakistan [121,122]. Moreover, acute exposure to 50 °C for as little as 5.5 h is sufficient to impair germination and prevent emergence entirely [123]. Heat stress inhibits seed germination through multiple mechanisms (Figure 3A). It reduces the stability and fluidity of the cytoplasmic membrane, thereby weakening seed germination rate and vigor [124]. Elevated temperatures also significantly decrease mitochondrial ATP levels in seeds, disrupting starch synthesis and protein composition, which in turn reduces seed vigor [125]. Under heat stress, intracellular enzymes may denature, preventing seeds from effectively breaking down stored materials and converting them into energy required for embryo development, thereby hindering normal germination [123]. Research indicates that under a maximum diurnal temperature of 40 °C, cotton cultivars with higher seed weight showed the least impairment and achieved greater germination and emergence rates than smaller-seeded cultivars [123], which could be due to the larger accumulation of storage substances in higher-seeded cultivars, thereby providing more energy to support germination. Moreover, heat stress can lead to abnormal accumulation of ABA within seeds, further inhibiting germination [126]. Excessively high temperatures accelerate transpiration as well, reducing water availability to seeds and slowing germination [126]. Thus, heat stress reduces germination and emergence rates through multiple mechanisms, which may include reduced membrane stability and fluidity, disruption of carbon metabolism balance, enzyme denaturation, ABA accumulation, and exacerbated water loss, which ultimately impair later development and reduce cotton yield.

### 4.2. Mitigation Strategies and Knowledge Gaps

Improving the germination ability of cotton seeds under high-temperature conditions through cultivation techniques has become a critical buffer against climate-driven yield losses. Recent studies have shown that plants can develop within-generation heat stress memory, whereby brief and moderate heat exposure enhances subsequent tolerance to high temperatures [127,128,129,130,131] (Figure 3B). This memory mechanism involves pathways such as protein modification and transcriptional regulation [127], and may even be transmitted as stable transgenerational inheritance [132], thereby improving seed survival under harsh environments. Consequently, short-term seed acclimation protocols could be optimized as a pre-sowing conditioning strategy to prime germination resilience in cotton under un-predictable thermal environments. In addition, exogenous substance applications are an effective strategy for improving cotton seed germination under heat stress. For example, Chakma et al. (2021) reported that 24-epibrassinolide (EBR) significantly alleviates the inhibitory effects of salt and heat stress on cotton seed germination, counteracts the negative regulation by ABA, and promotes seedling growth [133]. Lauxen et al. (2016) found that thiamethoxam treatment improves cotton emergence rates under high temperatures (35 °C) [134]. Nevertheless, the germination stage of cotton remains highly sensitive to temperature, and current research on the physiological, biochemical, and molecular regulatory mechanisms under heat stress at this stage is still severely insufficient. Therefore, to address the increasing frequency of extreme heat events under global warming and ensure stable cotton yield and quality, there is an urgent need to systematically elucidate the molecular mechanisms underlying heat stress responses during germination, identify key heat-tolerant genes, and promote the selection and utilization of resistant varieties.

## 5. Advances in Cold-Stress Resistance During Cotton Seed Germination

Cotton is a thermophilic crop that originated in tropical and subtropical regions and remains sensitive to low temperatures despite long-term domestication [135]. Driven by national food security strategies and rapid agricultural development in Xinjiang, China’s cotton cultivation has increasingly shifted toward the inland northwest in recent years. Long-term production experience and recent studies indicate that agrometeorological hazards such as frost, hail, strong winds, and sandstorms pose serious threats to cotton yield and quality in Xinjiang. Among these, chilling damage during the growing season, particularly the late-spring cold phenomenon, represents one of the most severe natural constraints on cotton production [136]. Germination is one of the most temperature-sensitive stages in cotton development; seeds can only initiate germination above a critical temperature threshold. Germination slows markedly below 20 °C and becomes severely inhibited or even completely arrested at 12–15 °C [137,138]. Given this stage’s extreme vulnerability to low-temperature stress and the direct impact of cold tolerance on seedling establishment, elucidating cold-tolerance mechanisms during cotton germination addresses both a fundamental scientific question in crop stress biology and an urgent practical need for stabilizing and expanding production in Xinjiang’s cotton region [139].

### 5.1. Damages of Cold Stress on Cotton Germination

**Figure 4:**
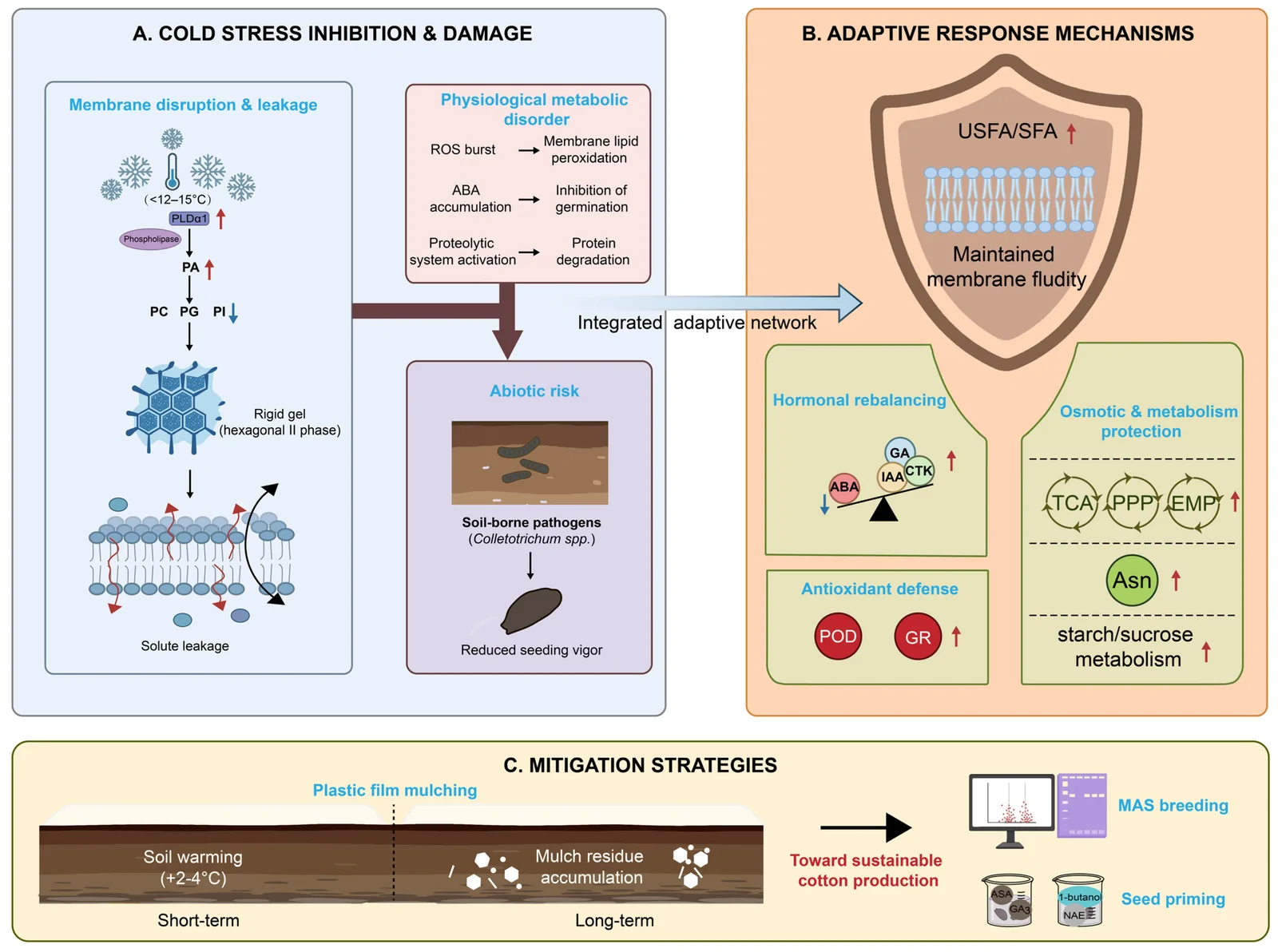
Schematic overview of cold stress impacts and mitigation strategies for enhancing cotton seed thermotolerance. (**A**) Cold stress suppresses cotton seed germination by inducing membrane phase transition and rigidification, ROS overaccumulation, ABA-dominant hormonal imbalance, and protein catabolism, while increasing susceptibility to pathogenic infection. PA, phosphatidic acid; PC, phosphatidylcholine; PG, phosphatidylglycerol; PI, phosphatidylinositol. (**B**) Cotton seeds maintain cellular homeostasis under cold stress by integrating membrane fluidity regulation, hormonal rebalancing, enhanced antioxidant defense, and metabolic protection to ensure successful germination. USFA, unsaturated fatty acids; SFA, saturated fatty acids; GR, glutathione reductase; TCA, tricarboxylic acid; PPP, pentose phosphate pathway; EMP, glycolysis; Asn, asparagine. (**C**) Integrated strategies for enhancing cold tolerance, encompassing cultivation management, seed priming, and molecular breeding technologies. ASA, acetylsalicylic acid; GA, gibberellic acid; NAE, N-acylethanolamine. Red arrows indicate upregulation/enhancement, and blue arrows indicate downregulation/attenuation.

Low temperatures inhibit cotton seed germination and seedling growth mainly through three interconnected mechanisms (Figure 4A). Membrane phase transition and imbibitional chilling injury constitute the primary target. Low temperatures shift membrane lipids from a fluid liquid-crystalline state to a rigid gel phase, especially during the vulnerable imbibition phase [140,141], leading to solute leakage. Recent studies further reveal that cold stress increased phosphatidic acid (PA) alongside decreased phosphatidylcholine (PC), phosphatidylglycerol (PG), and phosphatidylinositol (PI) promotes a leaky hexagonal II membrane arrangement, further exacerbating solute loss and germination inhibition [142]. Oxidative damage and hormonal imbalance represent another key pathway. ROS burst-triggered membrane lipid peroxidation [143,144] and ABA-mediated germination suppression [143] act in concert to inhibit germination, suggesting that coordinated regulation of antioxidant systems and ABA signaling pathways may represent a critical node for breaking through cold-tolerance bottlenecks. Metabolic disruption and heightened biotic stress risks comprise the third mechanism. Chilling activates proteolytic systems that degrade storage and photosynthetic proteins, weakening seedling vigor [145], and prolonged exposure to low temperatures extends the seed’s residence in soil, increasing susceptibility to soil-borne pathogens such as *Colletotrichum* spp., thereby further reducing germination rates [146]. Low temperature markedly suppresses radicle growth and development during the post-germination cotyledon stage in cotton [41] by attenuating hypocotyl elongation [147] and inducing sprout rot and other developmental abnormalities [136], thereby delaying flowering and fiber maturation and ultimately reducing yield and quality [148]. While some mechanisms underlying low-temperature inhibition of cotton germination are recognized, the interplay among these factors and the integrated regulatory networks involved warrant deeper investigation.

### 5.2. Response of Cotton Seed to Cold Stress at Germination Stage

To counteract the negative impact of low temperature on sowing and seedling establishment, cotton seeds have evolved an integrated network that couples physiological adjustments with molecular regulation (Figure 4B). Central to this adaptive strategy is the increase in the unsaturation level of storage fatty acids during early imbibition, as the cis-double bonds of unsaturated fatty acids introduce kinks that lower melting points relative to saturated homologues. These low-melting unsaturated fatty acids are preferentially mobilized at suboptimal temperatures, ensuring rapid energy provision for embryonic growth, while simultaneously preserving the fluidity of phospholipid bilayers that house respiratory and signaling proteins, an attribute indispensable for germination under chilling stress [140]. Consequently, genotypes exhibiting an elevated unsaturated fatty acids/saturated fatty acid ratio display superior cold tolerance at the germination stage [140,149]. Shim et al. (2019) [150] further demonstrated that fatty acid mutants with higher linoleic acid (58.20–61.80%) and lower palmitic acid (17.30–19.60%) content exhibited enhanced germination indices at 12 °C. Given the significant difference in melting points between the two fatty acid chains, the reduced proportion of palmitic acid effectively lowered the cumulative melting point of fatty acids in the mutant seeds. This promotes faster breakdown of lipid reserves under low temperatures, supporting seed germination [150], suggesting that optimizing the saturated/unsaturated balance offers a tractable target for cold-resilience breeding. In addition, suppression of phospholipase-mediated PA production mitigated membrane leakage and further improved seedling emergence in chilled cotton seeds [151].

Simultaneously, they activate three integrated modules encompassing antioxidant defense, hormonal rebalancing, and osmotic protection (Figure 4B). Transcriptomic profiling of the chilling-tolerant line KN27-3 revealed strong up-regulation of genes encoding both antioxidant synthesis and antioxidant activity, rapidly suppressing the oxidative burst. Hormonal governance is simultaneously rewritten, with IAA, CTK and GA biosynthetic transcripts surging, ABA signaling being muted, and the radicle being released from dormancy lock. At the energy level, transcript abundance of core enzymes in glycolysis, the tricarboxylic acid cycle and the oxidative pentose–phosphate pathway rose in parallel, securing sustained ATP and carbon-skeleton supply; up-regulation of asparagine synthetase genes further drove rapid accumulation of compatible solutes, lowering osmotic potential, limiting chilling-induced water loss and stabilizing the water status required for endosperm mobilization [143]. In another tolerant cultivar, Xinluzao 25, Hu et al. (2023) [144] reported pronounced induction of antioxidant enzyme genes together with enrichment of phenylpropanoid biosynthesis genes that modulate POD activity via phenylalanine and cinnamic acid. Enhanced cysteine and methionine metabolism provided substrates for glutathione, establishing a secondary antioxidant loop, while high expression of starch/sucrose-metabolism genes ensured a continuous supply of soluble sugars that serve both as energy and signaling molecules [144]. Zhang et al. (2024) imposed chilling stress at the late-germination, unexpanded-cotyledon stage and demonstrated that cold-tolerant genotypes alleviated growth inhibition through elevated antioxidant enzyme activities and higher endogenous IAA, CTK and SA levels [40]. Intriguingly, 100-seed weight correlates positively and heritably with germination-stage chilling tolerance, offering a simple, high-throughput auxiliary trait for early generation selection [152]. Collectively, membrane lipid remodeling, antioxidant–hormone coordination, and energy–osmotic adjustment constitute an integrated molecular–physiological strategy enabling cotton seeds to withstand chilling stress and establish viable seedlings. However, current understanding remains largely descriptive, with three critical gaps limiting application: (i) the signal transduction cascade linking membrane rigidification to transcriptional reprogramming remains uncharacterized; (ii) the relative contribution of each module to overall tolerance across genetic backgrounds is unquantified; and (iii) the efficacy of these mechanisms under field-relevant fluctuating low temperatures versus constant laboratory chilling is untested. Closing these gaps requires multi-omics integration and quantitative trait locus mapping of module-specific components to dissect the genetic architecture of integrated cold tolerance. Future research should prioritize constructing a membrane stability-redox homeostasis-energy metabolism trinity evaluation system for cold tolerance, alongside developing phenomics platforms capable of simulating field-fluctuating low temperatures. This would bridge the translational gap between basic research and breeding applications, delivering actionable adaptive solutions for cotton production in regions facing increasingly frequent extreme weather events under climate warming.

### 5.3. Strategies to Improve Cotton Seed Germination Under Cold Stress

To mitigate the low temperature suppression of cotton germination and emergence at sowing, producers routinely adopt temperature-borrowing agronomic practices. In early-maturing regions such as Uzbekistan and Xinjiang, seeds are almost always sown under transparent polyethylene film, which raises the topsoil temperature by 2–4 °C [153] (Figure 4C). By blocking long-wave radiation and reducing latent-heat flux, the film reshapes the soil micro-climate and simultaneously delivers a bundle of eco-economic benefits including weed suppression, moisture conservation, carbon sequestration, emission reduction and yield increase [108]. However, despite widespread mechanized retrieval efforts, a significant portion of polyethylene film persists in the soil due to incomplete removal and historical accumulation. Nationwide field surveys in China indicate that cropland plastic residue has reached 550,800 tons [154]. These persistent fragments progressively degrade soil architecture by increasing bulk density and reducing porosity, thereby restricting root elongation and compromising overall crop productivity [155,156]. This deleterious impact is strictly dose-dependent, with every incremental 100 kg ha^−1^ of film residue suppressing water infiltration by 8% and nutrient availability by 0.8–5%, leading to a consistent 3% reduction in final yield. Over time, the yield penalty from residual plastic eventually outweighs the initial gain from mulching [157]. This paradox of short-term climate adaptation creating long-term production constraints underscores an urgent need to decouple cold tolerance from plastic dependency through genetic solutions. The development of plastic-mulch-free cotton production systems has gained substantial momentum in China, driven by the urgent need to address plastic residue pollution in Xinjiang’s cotton belt [158]. Pioneering breeding efforts led by Yu and colleagues have successfully developed early-maturing germplasm (e.g., CCRI 619) with combined tolerance to cold, salt, and drought stresses, enabling yield levels of approximately 350 kg mu^−1^ under mulch-free conditions in southern Xinjiang [159], suggesting that genetic solutions can progressively replace agronomic interventions for sustainable cotton production.

Systematic identification of chilling-tolerance genes and regulatory networks in cotton, integrated into molecular-design breeding to create low-film-dependent, cold-resilient cultivars, is the essential route to breaking this bottleneck and achieving sustainable cotton production in northern China. However, progress in cotton cold-tolerance breeding remains fragmentary, confined largely to germplasm screening and genetic mapping without resolving underlying mechanisms. Shen et al. (2023) reported that emergence rate, dry weight, and total seedling length under low-temperature stress were associated with the locus *GhD09G0189* (*GhSAL1*) via GWAS, but its functional role during germination was not experimentally validated [135]. Shen et al. (2025) identified *GhD6PKL2* as a key candidate gene for cold stress tolerance during cotton germination via GWAS, and its silencing by VIGS reduced germination rate and hypocotyl elongation compared to the control under low-temperature treatment, though the underlying mechanism remains to be fully elucidated [160] (Table S4). Baytar et al. (2022) evaluated 110 genotypes under 18 °C germination stress and identified three molecular markers, BNL0569, CIR081 and CIR202, as candidate loci for marker-assisted selection [152]. Dhaliwal et al. (2024) identified two highly cold-tolerant cotton genotypes, FAM1 and FAM2, whose seeds achieved germination percentages of 81.97% and 60.00%, respectively, at 12 °C even without hydropriming treatment at 30 °C for 8 h. These genotypes thus represent potential donors for cold-tolerant breeding, although their genetic basis remains unclear [140]. More critically, functional validation of the relevant genes has so far relied almost exclusively on heterologous systems such as *Arabidopsis* (Table S1), leaving the native cotton mechanisms largely undefined and limiting translational applicability. To achieve a substantive breakthrough in cold-tolerance molecular breeding, it is essential to return to cotton itself, expand mapping populations and establish high-throughput phenomics platforms, thereby laying a solid theoretical foundation for the development of high-quality, cold-resilient cultivars.

In addition, various seed treatment technologies have been proven effective in improving cotton seed germination and seedling growth under low-temperature conditions. The application of exogenous priming agents has consistently been a favorable approach for enhancing cotton seed germination under cold stress (Figure 4C). For example, treatment with exogenous GA_3_ significantly increased germination rate and uniformity at 12–15 °C by regulating endogenous hormone balance (elevating the GA_3_/ABA ratio), enhancing antioxidant enzyme activity, and accelerating the metabolism of storage reserves [161]. Priming with antioxidants such as ascorbic acid (AsA) activates both enzymatic and non-enzymatic antioxidant systems, effectively scavenging ROS, reducing membrane lipid peroxidation, alleviating cold-induced germination inhibition, and promoting root development [162]. Meanwhile, osmotic priming agents like mannitol improve germination rate and uniformity at suboptimal temperatures (12–18 °C) by modulating seed water potential and metabolic activity, thereby reducing the accumulated thermal time required for germination [163]. Most recently, phospholipase inhibitors (1-butanol and NAE) have emerged as a mechanistically distinct strategy, alleviating PA accumulation-induced membrane damage, reducing electrolyte leakage under cold stress, and thereby improving seed germination performance under low-temperature conditions [142].

Currently, the genetic architecture of cotton cold tolerance remains essentially uncharted. No causal genes have been functionally validated in cotton itself; existing markers (BNL0569, CIR081, CIR202) and donor lines (FAM1, FAM2) await mechanistic characterization. Given the central role of storage lipid remodeling, evidenced by the tight correlation between unsaturated fatty acid ratios and suboptimal-temperature germination, future efforts should prioritize dissecting the regulatory pathways governing lipid unsaturation, mobilization kinetics, and membrane phase behavior to support breeding efforts. Such research needs to leverage these phenotypic anchors to translate quantitative trait variation into editable molecular targets. Only through systematic gene identification and native-species validation can molecular-design breeding deliver low-film-dependent cultivars, breaking the current lock-in to plastic mulching. While genetic improvement provides stable, heritable tolerance, its multi-year development cycle necessitates interim solutions; seed priming offers rapid, flexible protection but lacks persistence across generations and requires rigorous evaluation of potential carryover effects on fiber development and yield. Ultimately, breaking the plastic-dependency bottleneck requires reimagining cold tolerance as a dynamically optimized trait, iteratively refined through feedback between laboratory gene discovery, controlled priming protocols, and multi-location field validation under fluctuating thermal regimes.

## 6. Conclusions and Future Perspectives

Against a backdrop of accelerating climate change and increasingly frequent weather extremes, improving germination-stage tolerance is vital for securing uniform emergence, robust seedling establishment and final yield of cotton, the world’s dominant fiber crop. Insight into these mechanisms is indispensable for stabilizing global cotton supply and for easing competition between food and fiber for limited arable land. Germination is a critical stage at which cotton is severely affected by abiotic stress [164]. Salinity, drought and temperature extremes inhibit water uptake, disturb ABA/GA balance, trigger reactive oxygen species bursts, damage membrane integrity and function, disrupt energy metabolism and heighten vulnerability to pathogens, collectively reducing germination percentage and seedling quality. Nevertheless, cotton seeds have evolved both stress-specific and convergent protective strategies. Under salt stress the seed mobilizes osmotic adjustment, ion sequestration, antioxidant enzymes and ABA/GA signaling; quantitative trait loci and key regulators such as *GhSnRK2.6* and *GhCIPK6a* have been identified. During drought, plastic root architecture, accumulation of compatible solutes, activation of antioxidant defense and transgenerational stress memory enhance survival, with *GhADF1* and *GhMPK7* emerging as important molecular nodes. Cold adaptation is closely linked to increased membrane-lipid unsaturation, elevated antioxidant capacity, hormonal re-balancing and maintained energy metabolism. Intriguingly, membrane lipid remodeling emerges as a convergent mechanism across cold and drought responses, reflecting shared biophysical constraints on cellular membrane integrity. This convergence suggests that lipid unsaturation pathways could serve as multi-stress breeding targets. By contrast, the physiological and molecular mechanisms of heat stress on cotton germination remain essentially uncharacterized, a gap that is particularly urgent given global warming trends.

Mitigation strategies require hierarchical deployment. Traditional agronomic practices include mulching, which raises topsoil temperature by 2–4 °C but causes soil degradation and yield losses through residual plastic pollution, and precision irrigation scheduling that partially alleviates salt/drought stress. These measures manage the environment around the seeds but do not enhance the seed’s intrinsic capacity, rendering them vulnerable to climatic unpredictability and input cost inflation. Seed priming serves as an interim technology, rapidly enhancing germination resilience through hydropriming, osmopriming, hormonal priming, or biopriming, yet its pleiotropic risks are underappreciated. Notably, these treatments impose temporary physiological adjustments rather than heritable adaptations, raising concerns about potential carryover effects on fiber development, boll retention, and reproductive-stage stress resilience. Furthermore, their efficacy and safety margins depend on seed lot quality, environmental conditions, and climatic variability, requiring continuous recalibration and rigorous batch-specific validation. Consequently, priming is best understood as a bridging technology, valuable for risk reduction during cultivar development, but not a substitute for genetic advancement. The fundamental solution lies in genetic improvement. Breeding offers the best pathway to combine stress tolerance with yield potential in a heritable, self-propagating form. Crop breeding has progressed from empirical selection (Breeding 1.0) to artificial-intelligence-driven smart breeding (Breeding 5.0), with marker-assisted selection, genomic editing exemplified by CRISPR/Cas9, and multi-omics platforms providing powerful tools for pinpointing stress-resistance genes and for creating new cotton cultivars with superior germination resilience and high yield. Notably, enhancing stress tolerance may incur potential negative trade-offs with yield potential [165,166]. In most cases, plants rely on sophisticated regulatory mechanisms to dynamically switch between “growth mode” and “survival mode” in response to the energy status of their environment [165]. Nevertheless, accumulating evidence suggests that such trade-offs are not immutable. For example, transgenic cotton over-expressing *AtEDT1*/*HDG11* has demonstrated improved tolerance to both salt and drought stresses, concomitant with increased yield under water-deficit conditions [166].

Although significant progress has been made, several critical issues remain to be thoroughly investigated. Therefore, future research should focus on the following directions. First, functional characterization of stress-related genes during cotton germination still heavily relies on model plants such as *Arabidopsis*; thus, validation in cotton through genetic transformation and gene editing is urgently needed to clarify their roles within cotton-specific regulatory networks. Simultaneously, large-scale mining of natural variation for stress tolerance during germination in cotton germplasm resources should be conducted to identify superior alleles. Second, the transition from dry seed imbibition to radicle protrusion involves several key physiological switching points; high temporal resolution omics, live imaging, and controlled-environment simulations are required to dissect the spatiotemporal dynamics of osmotic sensing, calcium oscillations, hormonal balance, and energy metabolism, thereby generating a comprehensive roadmap from signal perception through transcriptional reprogramming and metabolic rewiring to phenotypic output. Third, transgenerational stress memory represents an important route to enhance progeny stress tolerance; future studies need to elucidate the specific roles of DNA methylation, histone modifications, and small RNAs in the formation, maintenance, and inheritance of stress memory during cotton germination, providing new targets for breeding smart cultivars with pre-adapted resilience. Fourth, field environments typically involve multiple simultaneous stresses such as combined salt and drought or high temperature plus drought, whose synergistic or antagonistic effects on germination are far more complex than single stresses; thus, research on physiological and molecular response networks under combined stresses needs to be strengthened to clarify their interaction rules. Fifth, integration of artificial intelligence including machine learning and deep learning, high-throughput phenomics especially real-time imaging during germination, large-scale genomic data, and environmental information should be used to construct genotype–phenotype–environment prediction models for cotton germination stress tolerance, analogous to the AI-driven approaches recently used in maize to identify candidate genes for nitrogen use efficiency (NUE) [167]; on this basis, model-driven molecular-design breeding strategies can be developed and intelligent context-specific seed treatments and sowing protocols can be designed to achieve precise regulation of germination and seedling establishment under adverse conditions. Through multidisciplinary, multilevel, and multi-technology research, a precise design and regulation system for cotton seed germination resilience can ultimately be established, providing solid theoretical and technical support for breeding high-yielding, high-quality, and highly-resistant cotton cultivars, optimizing stress management strategies, and enhancing the resilience and sustainability of global cotton production.

## Figures and Tables

**Table 1:** QTL loci related to salt tolerance during the cotton germination.

QTL/Gene	Chromosome	Material	Method	Reference
*qRGR-A04-1*	A04	RIL	QTL	[62]
*Loci-Chr4-2*	A04	RIL	QTL + RNA-seq	[58]
*Loci-Chr5-4*	A05			
*Gh_D01G0943*	D01	Natural population	GWAS	[63]
*Gh_D01G0945*				
*Gh_A01G0906*	A01			
*Gh_A01G0908*				
*Gh_D08G1308*	D08			
*Gh_D08G1309*				
*Gh_A01G1563*	A01	Natural population	GWAS + RNA-seq	[64]
*Gh_A02G1100*	A02			
*Gh_A03G1740*	A03			
*Gh_A07G0622*	A07			
*Gh_A07G0623*				
*Gh_A12G0877*	A12			
*Gh_D07G0243*	D07			
*Gh_D07G0249*				
*Gh_D07G0250*				
*Gh_D07G0251*				
*Gh_D07G0258*				
*Gh_D07G0263*				
*Gh_D07G0500*				
*qRSR-D07-01*	D07	Natural population	GWAS + RNA-seq	[65]
*qRSR-D11-01*	D11			
*GH_D06G0273*	D06	Natural population	GWAS + RNA-seq	[66]
*GH_A10G2036*	A10			
*GH_D06G0283*	D06			

## Data Availability

No new data were created or analyzed in this study. Data sharing is not applicable to this article.

## Supplementary Materials

The following supporting information can be downloaded at: https://www.mdpi.com/article/10.3390/plants15182881/s1, Table S1: Overview of cotton stress responsive genes validated through heterologous expression in heterologous receptors [30,168,169,170,171,172,173,174,175,176,177,178,179,180,181,182,183,184,185,186,187,188,189,190,191,192,193,194,195,196,197,198,91,199,200,201,202,203,204,205,206,207,208,209,210,211,212]; Table S2: Functional characterization of candidate genes in cotton under multiple abiotic stresses (salt, drought, heat, and cold) at the germination stage [41,54,56,88,92,160]; Table S3: Overview of various priming agents and their effects on mitigating salinity-induced inhibition in cotton seeds [45,46,47,133,213,214,215,216,217,218,219,220,221,222,223,224,225,226,227,228,229,230,231,232,233,234,48,235,236,237]; Table S4: QTL loci related to drought, heat, and cold tolerance during the cotton germination [99,135,160].
